# A review of the essential visual skills required for soccer: Beyond 20–20 optometry

**DOI:** 10.3389/fspor.2022.965195

**Published:** 2022-10-12

**Authors:** Lourens Millard, Gerrit Jan Breukelman, Nonkululeko Mathe, Ina Shaw, Brandon S. Shaw

**Affiliations:** ^1^Department of Human Movement Science, University of Zululand, KwaDlangezwa, South Africa; ^2^School of Sport, Rehabilitation and Exercise Sciences, University of Essex, Colchester, United Kingdom

**Keywords:** visual skills, sport vision, soccer vision, vision in sport, perceptual skills

## Abstract

In ball sports such as soccer, the visual system is critical in guiding a player's search for crucial information that underpins skillful behavior, which requires the incorporation of all of the relevant information in the environment in order to make successful decisions under pressure. However, vision in sport, and focusing on the specific visual skills required to be successful in a particular sport has largely been a practice ignored by experts and coaches as being an essential component of athletic performance. This is the first attempt to summarize and compile the necessary visual skills for soccer. This review's evidence suggests that, while current research still tends to focus on visual skills as a whole, there is a need to streamline this focus to the necessities of a particular sport. Furthermore, in identifying the visual skills essential for soccer, it allows for the effective training and testing of these skills, as well as for talent identification.

## Introduction

Vision is the signal that guides the body to respond and provides information to athletes on when and where to act (1). Athletic Performance may suffer if the vision system does not receive the message correctly or quickly enough. It is important for the visual system to function at a high level because athletic performance is one of the most demanding activities for the visual system (2). Vision is much more than seeing clearly, it is interconnected visual skills which affects performance. Just as exercises and drills can improve speed and strength, it can also improve the athlete's visual fitness and visual accuracy (3). In sport, vision may affect athlete's performance, including visual clarity, athletic performance (the ability to perform specific tasks) and information processing. The general ability to process and respond to visual stimuli also greatly improves the athlete's visual ability (2).

The science of improving visual skills to assist athletes in achieving the highest performance levels are becoming increasingly important in training many sports. Sport science performance analysis has undergone considerable changes recently, mainly due to the increased access and application of improved technology in computer science (3). Vision tests and sports training can help athletes determine the function of the eyes, beyond a basic ability to clearly see the letters and objects on standard eye charts (4). By discovering if there are weaknesses in these areas, coaches may have the opportunity to assist athletes to improve these visual skills, as well as the results produced in sports (5). Athletes who make full use of the visual system will gain the best possible performance levels, as well as a competitive advantage. By discovering whether there are weaknesses in these areas, coaches can have the opportunity to help athletes not only improve these visual skills, but also improve the athlete's performance in sports (5). Athletes can devote thousands of hours of physical training to improve their physical fitness; however, if vision or visual processing ability is insufficient, physical training may not be optimized and athletic performance may be affected (6). Athletes who make full use of the potential of the visual system will gain optimal levels of performance (3).

Soccer has successfully attracted the attention of global audiences and is currently the most popular sport in the world (7). Controlling the ball on the ground requires accurate motor and visual skills in order to keep opposing players away from the ball and advance it from one end of the of pitch to the next (8). Soccer requires various explosive activities, including jumping, kicking, positioning, turning, and running, changing pace, and maintaining a strong contraction to balance the ball at player's feet (9). To enable soccer players to perform the necessary skills affectively, visual skills are essential (10).

Reading a soccer game requires the player to maintain a constant vision of his or her surroundings. This is a special skill, that's hard to train. With soccer vision, the player sees the next phase of the game before others (11). Competitive soccer players know that to reach a higher level requires practice and adjustment (12). Well-trained players focus on game preparation, but they also have what ordinary players lack: excellent visual skills and excellent timing and athletic skills (13). Soccer vision enables players to discover teammates and opportunities within a fraction of a second, and make decisions to change the game. Thankfully, the eyes can also be trained just like other parts of the player's body (14). Through the football vision training program, ordinary players can acquire the visual skills needed to enter the next level of competition (1). However, to effectively train or test soccer visual skills, one must figure out which of these skills are essential for soccer and future soccer playing superstars.

Over the years, the role of visual performance factors in soccer has received considerable attention, but many athletes still do not have access to evaluation and improvement methods (7). Although there are other studies that provide information about the visual skills essential for soccer, no study has created a comprehensive list of visual skills essential for optimal performance thus this type of review is necessary to understand this field of research. In this vain, this review article aims to create a comprehensive list of essential visual skills for soccer players to aid in not only knowing which visual skills needs to be trained, but will also aid in the development of a sport-specific Visio-Spatial Skill (VSS) test battery and identifying future talent.

## Methods

### Search strategy

**Figure 1:**
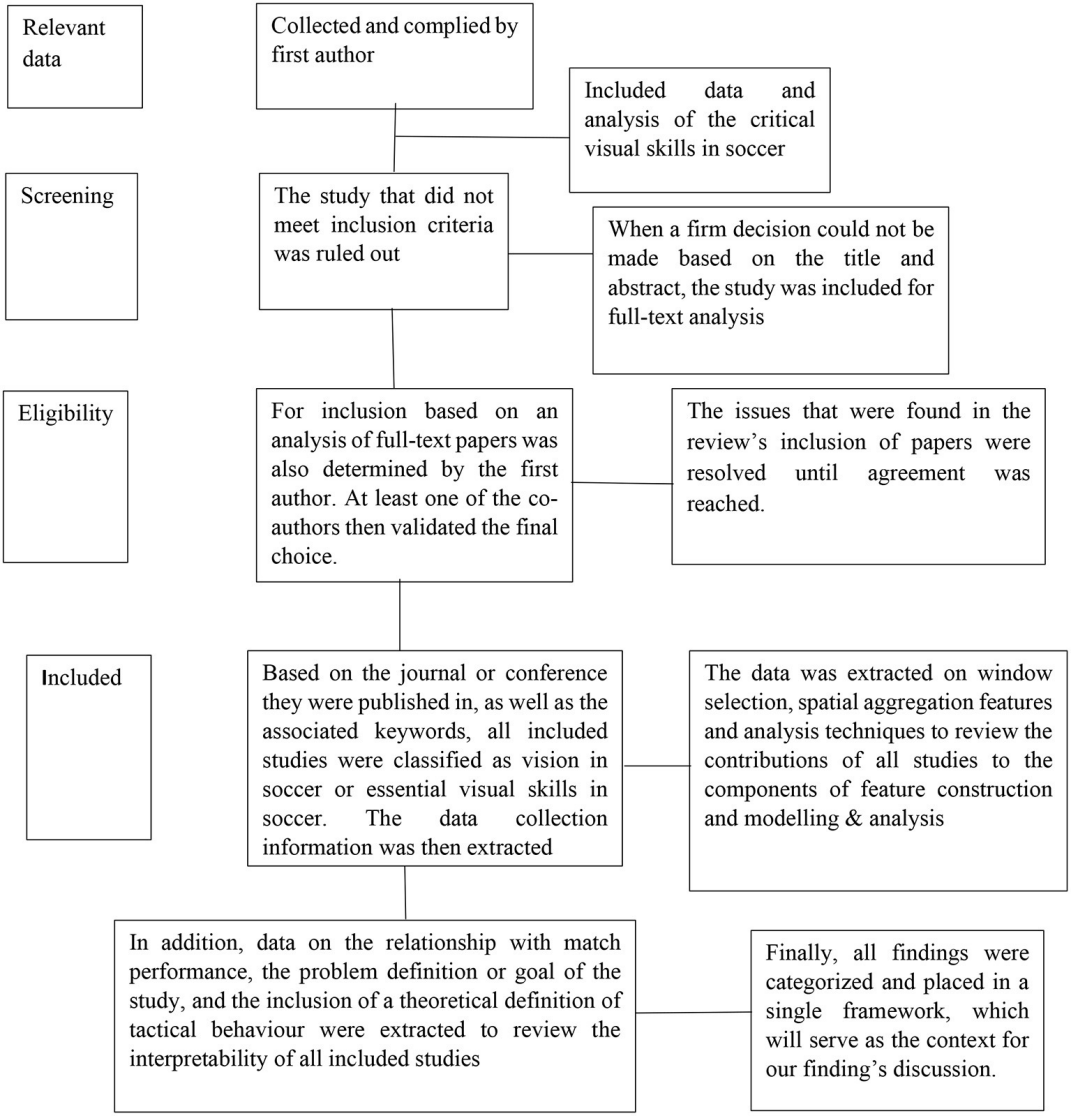
Data extraction.

An electronic search was conducted on the following databases to review the scholarly literature related to the visual skills required for soccer: Sport Discuss (1975–June 2021) EBM Reviews, PubMed (1996–June 2021), Current Contents, Science Direct, CISTI Source (1993–June 2021), Cochrane Database of Systematic Reviews, Google Scholar, and international e-catalogs. A keyword search yielded MeSH headings: “visual skills,” “sport vision,” “soccer vision,” “vision in sport,” “depth perception,” “eye-coodination,” “concentration in sport,” “fixation skill,” “focusing in sport,” “speed in soccer,” “reaction time,” “color discrimination,” “fusion flexibility,” “visual memory,” “contrast sensitivity”; which were fused and exploded. The searches were limited to peer-reviewed articles written in English. For discussion, original articles were identified and grouped. Figure 1 below illustrates the data extraction process.

## Results

This study used 105 full-text English-language papers from 150 citations found through electronic searches. Sixty-three Articles remained after removing duplicates and reviewing the full-text versions.

## Discussion

Vision is said to be the most variable of all the senses that influence athletic performance (15). The perception of visual information prevails over that of other sense systems, and it is considered essential to the proper performance of practically all sports (16). The use of imaging and visualization techniques is necessary for this sport. These skills aren't well-known in sports vision, though. Visual representations of soccer include non-static, meaning that the task calls for processing continuously changing visual input while it is in motion; persistent, requiring more than an hour of eye focus; dynamic, the player must continue to analyse the game (17). Soccer has developed into a sophisticated sport because of the numerous skills that need to be acquired. Despite the challenge of maintaining balance in the movement, the field of vision of the movement is constantly interrupted, and involves distance vision, direction positioning and spatial vision recognition (2).

Even though visual demand varies depending on the sport, some visual skills are essential in almost every sport (17). Because vision is the primary sense used in sports, it appears especially important to identify the visual skills required by athletes and to apply appropriate techniques to optimize them. Although previous studies looked at the impact of vision on performance, few, if any, of these studies also looked at outcomes, movement, and cognitive factors. Visual skills are divided into two categories: visual hardware (Table 1) and visual software (Table 2). The physical differences between a person's visual system's mechanical and optometric components are known as visual hardware (60, 65, 66). The cognitive variations that occur in the analysis, choice, coding, and general management of a person's visual input during a competition or in training are known as visual software (67, 68). All visual skills discussed in this review will be categorized according to whether they meet the criteria to be seen as visual hardware or visual software skills. The visual skills identified in this review is as follows: Visual Hardware: Visual acuity, Color discrimination, Contrast sensitivity, Eye focusing, Fusion flexibility, Fixation, Depth perception and Peripheral vision. Visual Software: Eye tracking, Concentration, Eye foot coordination, Speed & Span of recognition, Eye-body coordination, Visual adjustability, Visual reaction time and Visual memory.

**Table 1:** Essential visual hardware skills for soccer players.

**Visual skills**	**Description**	**References**
Visual acuity	Visual acuity is a measurement of an athlete's ability to resolve fine detail.	(2, 9, 18–20)
Color discrimination	Color discrimination is essential to identify team members and opponents during game.	(20, 21)
Contrast sensitivity	Contrast sensitivity is the individual's ability to detect low-contrast objects of various sizes.	(22)
Eye focusing	Eye focusing is the capacity to switch focus quickly and precisely between objects at various distances.	(23, 24)
Fusion flexibility	The capacity to combine the pictures that each eye processes into a single view.	(25)
Fixation	The ability to focus one's vision or judgment on a moving object during play may allow players to react more quickly and efficiently.	(1, 26–28)
Depth perception	Depth perception is essential for players to correctly identify where another player is or to judge the flight of aerial balls.	(20, 29–31)
Peripheral vision	Allows athlete to react to stimuli outside their normal central vision.	(6, 32–38)

**Table 2:** Essential visual software skills for soccer players.

Eye tracking	Eye movements are necessary to gather precise visual data from pertinent scene locations, enabling the best possible control of human motion in sports and active living.	(20, 39–44)
Concentration	This visual skill allows the player to focus on the game, specifically when spectators are reacting or cheering around the player.	(4, 45, 46)
Eye-foot coordination	Foot-eye coordination is a skill that enables players to dribble, make accurate free kicks, misdirect the opposition, and make exquisite passes	(47–49)
Peripheral vision	Allows athlete to react to stimuli outside their normal central vision.	(6, 32–38)
Speed and span of recognition	This visual ability is essential to quickly scan scene to absorb all the information in front of the player to react quickly to the situation.	(2, 4, 6, 50–53)
Eye-body coordination	Coordination is the ability to execute a series of movements smoothly and accurately repeatedly.	(54–57)
Visual adjustability	Visual adjustability, which refers to the ability of the eyes to integrate with the body, is the ability of the body to adjust motor movements in response to a stimulus.	(55, 56)
Visual reaction time	Reaction time refers to the capacity to act quickly, with appropriate stance and control, in response to a stimulus like sound or light	(2, 58–62)
Visual memory	Visual memory is the capacity of the eyes and brain to identify patterns on the field or court and to process that information swiftly and effectively	(18, 63, 64)

### Visual hardware

The capacity to distinguish the fine details of a moving object when the item and the observer are in relative motion is known as dynamic visual acuity (DVA). Visual acuity determines the players ability to see clearly while objects are on the move. The act of seeing in motion necessitates a different and more demanding visual acuity than viewing a stationary object (18). Visual acuity is a measurement of an athlete's ability to resolve fine detail. Additionally, it has been demonstrated that with training, dynamic visual acuity can be enhanced (19). Lack of visual acuity affects one's capacity to discern small items and see them clearly (20). According to research, athletes' greater DVA reflects their improved ability to track moving objects by making appropriate saccadic eye movements (9). Athletes must constantly maintain balance in the game, despite being constantly challenged in sports to maintain balance, the challenger must continue to drive the action in the game; and it involves long distance vision, positioning in the direction and visual recognition of the space (2, 19). Players with this visual ability will be able to clearly recognize a ball moving fast. This skill will also assist a player in maintaining sharp vision while the ball and other players are moving.

Color vision is the ability to distinguish between different colors and is caused by cone photoreceptors in the retina of the eye. These cones' light-sensitive pigments allow us to recognize colors (21). For soccer players, color discrimination is essential. This makes it easier for players to recognize their teammates and tell them apart from the referee or other players. It also makes it easier to identify the ball (20). Color discrimination is essential to identify team members and opponents during game, quick glare recovery can improve an athlete's performance, especially for night games played under artificial lights (21, 69). Players with this ability will be able to discern between a ball and other players in the game by noticing differences and similarities. This visual ability can be impaired, which could make it difficult to recognize differences, resulting in a decrease in performance.

Contrast sensitivity is the individual's ability to detect low-contrast objects of various sizes. A person's visual acuity is physiologically limited in three ways: optically, rationally, and cortically. An athlete's performance can be hampered by even a modest degree of residual refractive error. On the other hand, higher spatial frequencies are essential for the best visual performance (22). When there is insufficient light, fog, or glare the contrast between the object and its background tends to be reduced (22). Given that soccer players also play in difficult conditions and under different lighting levels, this visual ability will aid the players in immediately identifying and tracking the ball against a variety of backgrounds. When there is insufficient light, fog, or glare the contrast between the object and its background tends to be reduced.

Eye focusing is the capacity to switch focus quickly and precisely between objects at various distances. Eye focusing is necessary for athletes to be able to fixate fast on stable and clear vision, especially while trying to fixate from far to near or vice versa (23). Any sport that requires players to change their visual focus to goals at varying distances from them requires accommodation and vergence. Athletes' capacity for visual focusing s allows them to maintain a focused gaze on an item while it moves from one distance (near) to another (far) (23, 24). Focusing the eyes allows the player to see the stitches on the soccer ball and recognize corners or bike kicks, for a perfect timing response.

The capacity to combine the pictures that each eye processes into a single view is known as fusion flexibility. Players who have deficiencies may misjudge directions and distances during games and may experience double vision (25). With Fusion flexibility a player will quickly identify the ball as it moves in space (25). If there is a fusion deficit, a soccer player may have double vision, misjudge direction, and struggle to keep up with the ball and other players on the field of play. The entire athlete's performance is affected as a result therefore this skill is important to prevent that.

The Quiet eye (QE) is the last fixation or tracking gaze that is focused on a particular place or object within 3° of the visual axes for at least 100 ms. The QE can continue through and beyond the task's final movement because the offset occurs when the gaze deviates from the item or place by more than 3° of visual angle for a minimum of 100 ms before the commencement of the QE (1). According to Vickers and Williams (26), the process of focusing attention externally on crucial task information (through the QE) shields athletes from the anxiety's typically crippling effects. The ability to focus one's vision or judgment on a moving object during play may allow players to react more quickly and efficiently (27). Furthermore, it can facilitate the athlete's match preparation by allowing him or her to apply newly acquired ideas to their sport after learning them (27, 28). Expert athletes' longer fixation duration has typically been interpreted as experts extracting more information from each fixation. Shorter fixation durations, on the other hand, have been linked to more efficient visual information processing (as predicted by the theory of long-term working memory (27). Although these findings appear to be contradictory, they can be explained by the fact that gaze behavior is heavily influenced by environmental and task constraints. Overall, expert performers appear to use a more effective and task-specific gaze strategy (28). With this skill, a player will be able to transfer their attention from the ball to the other players despite the constant movement of their eyes. The player will have enough time and space during a penalty kick or free kick to focus on a certain area of the ball.

In soccer, depth perception is the most important predictive skill. Referees adjust the location of the ball during the game based on their anticipation and move around the field to get the best view of the game and the ball (29). To analyse the surroundings in three dimensions, depth perception is crucial (30). Depth perception is essential for players to correctly identify where another player is or to judge the flight of aerial balls (20). The most critical component of healthy vision is depth perception. The best stereopsis (depth perception) is necessary for an athlete to have the best stereo vision (31). Depth perception allows an athlete to estimate the relative distance between two objects that are at different distances from one another as well as to perceive movement in three-dimensional space. For an athlete to anticipate anything during any game, being able to estimate the target's distance and speed is crucial (31). Players may evaluate the distances between themselves, opponents, the ball, teammates, and boundary lines with speed and accuracy because to depth perception.

When a soccer player practices skills (kick, pass, run with a ball, and other techniques) during a match, it is important for him to have a good vision of the field and of the players with the goal of the player practicing a better action of the skill (32, 37). As a result, soccer players with an emphasis on peripheral vision must play with their heads up during the game because this action is essential for good soccer technique (38). Peripheral vison allows athletes to react to stimuli outside their normal central vision (6). A good peripheral vision can help players monitor their surroundings or keep their balance in team sports. For example, if a player wants to pass the soccer ball to a teammate, he should not look directly at his teammate and make a heel-dragging decision. Otherwise, he risks losing control of the ball because the defender will detect and prevent passing by detecting the opponent's eye gaze. As a result, the player must use his peripheral vision to gather information from the sports environment while remaining focused and not revealing his intentions to avoid defending actions by his opponents. If a player has better peripheral vision, he can notice his teammate earlier and make a better, more successful pass (33–36). Since much of what happens in a game does not occur directly, this skill will allow the player to notice action to the side without having to turn their head. This is especially helpful when a player spots a teammate out of the corner of his eye.

### Visual software

Eye movements are necessary to gather precise visual data from pertinent scene locations, enabling the best possible control of human motion in sports and active living (43). Soccer players must be able to monitor the ball with their eyes, which requires quick, accurate saccades (or eye movements) in order to keep up with the 21 other players and the ball on the pitch (39). Visualizing the relevant information as rapidly as feasible is crucial for making successful timely motion reactions to prior actions or judgments (44). In soccer, since the emphasis alternates between nearby and distant objects, eye tracking is crucial. Players almost never need to pay attention to things that are close to them when playing soccer. Additionally, the soccer ball is bigger and frequently moves at rates that reduce the need for eye tracking (20, 40). Soccer players must quickly and under pressure process a lot of internal and external information while also having to respond to complicated and constantly changing settings (41). During games, soccer players must divide their focus between several items (such as a moving ball, teammates, and opponents) (41, 42). Eye tracking is therefore a crucial indicator of a soccer player's performance. Players who possess this skill will be able to track a fast-moving ball and opponents without frequently shifting their heads, as well as maintain greater balance and respond to situations more swiftly.

Trying to decipher the correlation of the signal requires selective attention, particularly in a continually changing environment (45). All players may find that this is the ability that determines whether a championship is won or lost if they can learn to focus throughout a game while still taking advantage of an opponent's mistakes on the field (4, 46). Trainings that require the brain and body to work always can help improve concentration, focus, and attention span (46). Knowing when and how to shift attention during game play is another important aspect of concentration (70). This visual skill allows the player to focus on the game, specifically when spectators are reacting or cheering around the player. Trainings that require the brain and body to work always can help improve concentration, focus, and attention span (46). When players have concentration and good technique, the ball will easily go into the goal and the team will score. When a player has this visual ability, they won't be easily distracted and will perform at their best.

Soccer players must have good foot-eye coordination, which is widely known. Foot-eye coordination is a skill that enables players to dribble, make accurate free kicks, misdirect the opposition, and make exquisite passes. A player's ability to stop a soccer ball with his foot and adjust to intercept it is another benefit of having good foot-eye coordination. To keep his head up while handling the ball, a player needs to acquire foot-eye coordination (47). To make accurate shots that are completely on target and travel in the desired direction, one must have excellent eye-foot coordination (48). While field players or position players need exceptional eye-foot coordination to kick the ball correctly in the right direction. The players' feet move to follow the anticipated course as their eyes provide direction (49). This ability enables players to respond to incoming visual information with precise bodily movements. This ability is crucial for soccer because it allows exact timing and control over bodily movements, which are necessary for actions like heading the ball into the goal, preventing the ball from getting there, or going for that scissor kick at the right moment.

Fast visual processing speed has long been seen as a need for success in fast-action sport like soccer when discussing an athlete's ability to react quickly to visual cues on the field, we use the term “speed of recognition” (2). This visual ability is essential to quickly scan scene to absorb all the information in front of the player to react quickly to the situation (6, 50). Once the ball begins to bounce off the player struggling to control the ball, reaction speed may be the difference between winning the game or losing the fight (51, 52). A faster athlete, for example, may be able to reach a ball faster than an opponent or even outrun a pursuer. As a result, athletes in most sports place a premium on speed (53). A player should recognize the potential for a specific play development as soon as possible. Players have milliseconds to release a shot, make an accurate pass-through traffic, stop a shot, or identify an offensive or defensive set up (4). To decide whether to pass the ball, where to position himself next, and other play-related decisions, the player must be able to process a lot of information with only a fast sweep of the field.

Coordination is the ability to execute a series of movements smoothly and accurately repeatedly. The senses, muscular contractions, and joint movements may all be involved. Goalkeepers must have good eye-hand coordination to stop the ball from going through the goal posts (54). Poor coordination can lead to ineffective action performance (55, 56). For soccer players to monitor the ball in motion, win and hold possession of the ball, and transfer it to the goal area, eye coordination is crucial (57). Coordination issues lead to weakened skills, delayed change of direction, and poor body-on-body performances and poor passing techniques.

Visual adjustability, which refers to the ability of the eyes to integrate with the body, is the ability of the body to adjust motor movements in response to a stimulus. During a soccer game, the environment shifts, and the visual system needs to be adaptable enough to change motor responses swiftly and precisely. Lower visual adjustability slows down reactions, which leads to inconsistent skill execution (55, 56). Visual adaptability is especially useful in assisting players in dealing with difficult situations that may arise during a soccer game. The faster the player's visual system can adapt to changing needs during gameplay, the more predictable and consistent the player's movement response will be.

Reaction time refers to the capacity to act quickly, with appropriate stance and control, in response to a stimulus like sound or light. In games, it's more crucial to have quick reactions than to move quickly forward (58, 59). Faster visual processing and a shorter time for the neuromuscular system to transmit information to the muscles can both be the results of faster visual reaction time (2). Most of the time when playing, players must anticipate and react to the movement of their opponents (62). The quicker and more precise information an athlete can process can speed up decision-making and give them more time to prepare their motor behavior (71, 72). Reaction time (RT) and anticipatory skill are crucial components of perceptual abilities that have been deemed beneficial to the player's effective performance in sports domains (60). Individual variances will always exist, but any athlete can develop faster reactions (61). In soccer, maximal speed is rarely achieved or required, but an explosive reaction is a necessary requirement. The ability to tell who wins and loses depends heavily on the players' very little differences in reaction times (58, 59). The defensive player's success or failure in this scenario mostly depends on how quickly or slowly he reacts to the attacking player's move or faint (59). This skill will enable the players to move the ball fast, avoid clashing with other players, and react to the ball's shifting direction in a short amount of time.

Visual memory is the capacity of the eyes and brain to identify patterns on the field or court and to process that information swiftly and effectively. This is a skill that can be taught in and enhanced, making it a useful tool for athletes (18). Visual memory is crucial for the player to process and remember actions taking place during the game or after (63, 64). Compared to less talented opponents, skilled players are better at memorizing and recognizing play patterns, and they have a greater knowledge of what can happen in certain circumstances Players will be able to utilize this talent to record their gameplay and use the visuals they record to make quick, extremely accurate decisions in games.

## Conclusion

This review highlights the fact that although current research still tends to focus on visual skills, there is a need to streamline this focus to the necessities of a particular sport. Skill after skill, this review provides an in-depth list of not only the visual skills essential for soccer, but also the reason as why it leads to optimal performance. The sixteen essential visual skills for soccer identified in this review provides a starting point for adding additional visual skills. Furthermore, in identifying these skills, it allows future research to create visual skill test batteries that is specific to soccer, which in turn creates the opportunity to empirically prove whether these visual skills can be trained and lead to optimal performance. In providing the opportunity to test and train these visual skills it will allow coaches to identify talented players early, which in turn will lead to the possibility of competitive advantages.

## Author contributions

NM assisted with the design of the study, article dissection and formulation, and direction of the article. LM contributed the design of the study as well as the content of the study. BS and IS contributed with the article dissection, content of the study, writing as well as the formulation, and direction of the article. GB contributed the content of the study, writing as well as the formulation, and direction of the article. All authors contributed to the article and approved the submitted version.

## Conflict of interest

The authors declare that the research was conducted in the absence of any commercial or financial relationships that could be construed as a potential conflict of interest.

## Publisher's note

All claims expressed in this article are solely those of the authors and do not necessarily represent those of their affiliated organizations, or those of the publisher, the editors and the reviewers. Any product that may be evaluated in this article, or claim that may be made by its manufacturer, is not guaranteed or endorsed by the publisher.
